# Circulating p16-Positive and p16-Negative Tumor Cells Serve as Independent Prognostic Indicators of Survival in Patients with Head and Neck Squamous Cell Carcinomas

**DOI:** 10.3390/jpm11111156

**Published:** 2021-11-07

**Authors:** Pei-Hung Chang, Hung-Ming Wang, Yung-Chia Kuo, Li-Yu Lee, Chia-Jung Liao, Hsuan-Chih Kuo, Cheng-Lung Hsu, Chun-Ta Liao, Sanger Hung-Chi Lin, Pei-Wei Huang, Tyler Min-Hsien Wu, Jason Chia-Hsun Hsieh

**Affiliations:** 1 College of Medicine, Chang Gung University, Taoyuan 33382, Taiwan; ph555chang@gmail.com (P.-H.C.); whm526@cgmh.org.tw (H.-M.W.); 8705024@cgmh.org.tw (Y.-C.K.); r22068@cgmh.org.tw (L.-Y.L.); L329735@ms49.hinet.net (C.-J.L.); hsuanchihkuo@gmail.com (H.-C.K.); hsu2221@adm.cgmh.org.tw (C.-L.H.); liaoct@cgmh.org.tw (C.-T.L.); freewind05@cgmh.org.tw (P.-W.H.); 2 Division of Hematology-Oncology, Department of Internal Medicine, Chang Gung Memorial Hospital at Keelung, Keelung 20448, Taiwan; 3 Circulating Tumor Cell Lab., Division of Hematology-Oncology, Department of Internal Medicine, Chang Gung Memorial Hospital at Linkou, Taoyuan 33382, Taiwan; sangerhj@gmail.com (S.H.-C.L.); mhwu@mail.cgu.edu.tw (T.M.-H.W.); 4 Division of Hematology-Oncology, Department of Internal Medicine, New Taipei City Municipal TuCheng Hospital, New Taipei City 23600, Taiwan; 5 Department of Pathology, Chang Gung Memorial Hospital at Linkou, Taoyuan 33382, Taiwan; 6 Department of Otorhinolaryngology, Head and Neck Surgery, Linkou Chang Gung Memorial Hospital, Chang Gung University, Taoyuan 33382, Taiwan; 7 Tissue Engineering and Microfluidic Biochip Lab., Graduate Institute of Biomedical Engineering, Chang Gung University, Taoyuan 33382, Taiwan

**Keywords:** circulating tumor cells, p16 expression, head and neck squamous cell carcinoma, HPV genotyping, biomarker, liquid biopsy

## Abstract

Background: Decisions regarding the staging, prognosis, and treatment of patients with head and neck squamous cell carcinomas (HNSCCs) are made after determining their p16 expression levels and human papillomavirus (HPV) infection status. Methods: We investigated the prognostic roles of p16-positive and p16-negative circulating tumor cells (CTCs) and their cell counts in HNSCC patients. We enrolled patients with locally advanced HNSCCs who received definitive concurrent chemoradiotherapy for final analysis. We performed CTC testing and p16 expression analysis before chemoradiotherapy. We analyzed the correlation between p16-positive and p16-negative CTCs and HPV genotyping, tissue p16 expression status, response to chemoradiotherapy, disease-free survival, and overall survival. Results: Forty-one patients who fulfilled the study criteria were prospectively enrolled for final analysis. The detection rates of p16-positive (>0 cells/mL blood) and p16-negative (≥3 cells/mL blood) CTCs were 51.2% (*n* = 21/41) and 70.7%, respectively. The best responses of chemoradiotherapy and the p16 positivity of CTCs are independent prognostic factors of disease progression, with hazard ratios of 1.738 (95% confidence interval (CI): 1.031–2.927), 5.497 (95% CI: 1.818–16.615), and 0.176 (95% CI: 0.056–0.554), respectively. The p16 positivity of CTCs was a prognostic factor for cancer death, with a hazard ratio of 0.294 (95% CI: 0.102–0.852). Conclusions: The p16-positive and p16-negative CTCs could predict outcomes in HNSCC patients receiving definitive chemoradiotherapy. This non-invasive CTC test could help stratify the risk and prognosis before chemoradiotherapy in clinical practice and enable us to perform de-intensifying therapies.

## 1. Introduction

Human papillomavirus (HPV)-associated head and neck squamous cell carcinoma (HNSCC) has been widely investigated and thought of as a critical biomarker in HNSCC [1,2]. HPV-positive HNSCC, especially with oropharyngeal-originated tumors [3,4], has a significantly better prognosis (50% reduction of death risk) compared with those without HPV infection [5,6]. Even though patients with HPV-associated HNSCC had a better prognosis, primary concurrent chemoradiotherapy (CCRT) remains one of the standards of care [7]. De-intensification of CCRT has been widely investigated in very recent years [8,9]. HPV-positive tumors can also be found in patients with cancers originating in the head and neck region other than the oropharynx, such as the paranasal sinus [10], the hypopharynx [11], the larynx [12], and the oral cavity [13]. Given the positive prognostic impact of HPV infection, the p16 expression or HPV genotyping status at cancer staging have been strongly suggested for patients presenting with neck squamous cell carcinoma, without identified primary sites [14,15]. The importance of p16 expression or HPV infection status in HNSCC is well established [16].

P16 expression by immunohistochemistry staining is much easier to perform than PCR for HPV infection; therefore, p16 expression was much more widely used in clinical practices considering the test’s price and accessibility. However, some investigators reported that p16 expression does not always equal HPV infection [17]. HPV-DNA/RNA testing is still recommended for confirming p16 results, with increased specificity and diagnostic accuracy [17,18]. However, these tests are all tissue-based and require invasive procedures to obtain cancer tissue [17]. Sometimes, these invasive procedures caused unwanted complications, such as tumor bleeding [19]. However, there is no validated blood test for detecting HPV infection or evaluating p16 expression for diagnosis or monitoring, except for some exploratory or small-scale observational studies [20]. Only a few studies have reported technology detecting p16 expression levels in circulating tumor cells (CTCs) [21,22] or circulating tumor DNA [23,24] in plasma samples in HPV-associated HNSCC. The significance of p16 or HPV in peripheral blood remains unknown.

Therefore, we hypothesized the following: (1) that expressed p16 can be detected in CTCs; (2) that circulating p16-negative CTCs and p16-positive CTCs might have different effects on survival rates; (3) that p16 expression levels in the tissue and blood (CTCs) could be correlated with each other.

## 2. Materials and Methods

### 2.1. Patient Enrollment

Our prospective study was conducted in two medical centers, Chang Gung Memorial Hospital, Linkou, and Keelung, Taiwan. All patients provided written informed consent. The Institutional Review Board in Chang Gung Memorial Hospital approved the study protocols, with approval IDs 104-2620B, 103-7795B, and 201700867B0. Eligible patients with histologically- or pathologically- confirmed head and neck squamous cell carcinomas with p16 expression status were considered medically unfit for surgery or had surgically unresectable, locally advanced presentation (stage IIb–IV, American Joint Committee on Cancer [AJCC], 8th edition). In addition, patients with (1) age ≥20 years; (2) the ability to understand the protocol and provide informed consent out of their own free will; (3) primary HNSCC; and (4) adequate liver and renal function and white blood cell counts before undergoing anticancer therapies, especially chemoradiotherapy, were included. The exclusion criteria contained patients who (1) receive therapies except for CCRT, including curative surgery without CCRT, salvage surgery, or radiation alone; (2) refused to blood drawing in the protocol; (3) had rapidly worsened performance status to complete CCRT; or (4) had metachronous or synchronous double cancer. Physicians performed disease staging and management according to the standard treatment protocols detailed in institutional guidelines. Results were reported following the REMARK guidelines [25]. Examinations for the initial staging and response evaluation processes included magnetic resonance imaging and positron emission tomography. In accordance with standard treatment guidelines, concurrent chemoradiotherapy was scheduled and delivered by medical oncologists and radiation oncologists. In accordance with the guidelines of version 1.1 of the response evaluation criteria in solid tumors (RECIST), the treatment response was determined based on whether the patient exhibited complete remission (CR), partial response (PR), stable disease (SD), or progressive disease (PD). This was determined by the multidisciplinary head and neck cancer tumor board at Chang Gung Memorial Hospital.

### 2.2. Tissue Immunohistochemistry Staining for p16 Expression Analysis

Immunohistochemistry staining (IHC) was performed using a mouse monoclonal antibody against p16 (Roche E6H4™, catalog #725-4713) on a Ventana Benchmark LT automated immunostainer (Tucson, AZ, USA), per the standard protocol. Positive and negative controls were included routinely. A positive signal was defined as that obtained with nuclear and or cytoplasmic staining. If cells were stained via cytoplasmic staining alone, the result was considered negative. In this study, the positivity of tumor samples is defined by a result where ≥70% of cells are stained via cytoplasmatic and nuclear staining in two medical centers [26]. In this study, we performed HPV genotyping to confirm the HPV infection status in p16-positive cancer tissues (Supplementary Table S1).

### 2.3. The Isolation and Identification of Circulating Tumor Cells via Microscopy

Blood samples (8 mL for each patient, including 4 mL for microscopy and the other 4 mL for flow cytometry) were drawn before anticancer therapies, including chemotherapy or radiotherapy. A CTC enrichment procedure was performed by red blood cell lysis (by mixing 155 mM NH4Cl, 14 mM NaHCO3, and 0.1 mM EDTA in a 10:1 ratio with whole blood samples) and CD45-positive leukocyte depletion, using EasySep Human CD45 Depletion kits (Cat. NO. 18259, STEMCELL Technologies Inc., Vancouver, BC, Canada), following the manufacturer’s instructions. A previously detailed method for CTC enrichment and counting was used [27,28,29].

We further fixed CTCs, isolated from 4 mL of whole blood samples, using 4% paraformaldehyde (Cat. No. 15710, Electron Microscopy Sciences) for 10 min at 25 °C. Permeabilization was performed by treating cells with PBST (0.1% Triton X-100 in PBS) for 10 min at 25 °C. After washing cells with PBS, they were blocked with PBS containing 2% BSA and the HuFcR Binding Inhibitor (Cat. No. 14-9161-73, eBioscience, San Diego, CA, USA) for 30 min at room temperature. Before the antibody reaction, 0.0025% Trypan Blue (Cat. No. 15250061, Thermo Fisher Scientific, CA, USA) was added to block auto-fluorescence. The antibody reaction was allowed to occur upon the addition of anti-EpCAM antibody conjugated Alexa Fluor 488 (1:400, one hour, Cat. No. 5198, Cell Signaling, Danvers, MA, USA) and anti-p16 antibody conjugated Alexa Fluor 647 (Cat. No. ab199819, Abcam, Cambridge, UK). We used the Hoechst (Cat. No. 62249, Thermo Fisher Scientific, CA, USA) stain to stain the cell nucleus. Cell fluorescence images were captured using a fluorescence microscope (Zeiss Axioskop 2 Plus Fluorescence Microscope, Carl Zeiss Microscopy, LLC, United States; Leica TCS SP2 Confocal Laser Scanning Microscope, Leica Microsystems, Wetzlar, Germany). CTCs were defined as cells expressing EpCAM^pos^Hoechst^pos^CD45^neg^ and were further divided into p16-positive or p16-negative status.

### 2.4. Analysis of p16 Expression in Circulating Tumor Cells via Flow Cytometry

To determine the status of p16 expression in CTCs, we first fixed cells enriched via RBC lysis and CD45 depletion, using Fix & Perm Cell Permeabilization Reagents (Cat. NO. GAS003, molecular probes by Life Technologies, Thermo Fisher Scientific, CA, USA). Then, we added an anti-EpCAM antibody conjugated PE (Cat. No. FAB960P-100, R & D Systems) and an anti-p16 antibody conjugated Alexa Fluor 647 (Cat. No. ab199819, Abcam) during fixation and permeabilization. A secondary antibody, i.e., goat anti-mouse IgG H&L conjugated Alexa Fluor 488 (Cat. No. ab150113, Abcam), was also added to exclude the residual CD45-positive leukocytes (labeled with the CD45 antibody cocktail provided in the CD45 depletion kit). Isotype control antibodies were used as the negative control. After staining, cell samples were analyzed using a Flow Cytometer (CytoFLEXTM Flow Cytometer, Beckman Coulter, Inc., Pasadena, CA, USA).

Positive and negative controls for the analysis of p16 expression were carried out on circulating tumor cells.

As experimental controls for p16-positive cells, we used the HeLa (cervical cancer cells, ATCC^®^ CRM-CCL-2TM) cells as positive controls in CTC samples; whereas, HCT116 (colorectal cancer cells, ATCC^®^ CCL-247TM) and H1975 (non-small cell lung cancer cells, ATCC^®^ CRL-5908™) cells were used as negative controls to analyze p16 expression. We cultivated these cells per the instructions provided by the American Type Culture Collection (ATCC). Briefly, HeLa, HCT116, and H1975 cells were maintained in DMEM medium (Cat. No. 11965092, GIBCO), McCoy’s 5A medium (GIBCO), and RPMI-1640 medium (GIBCO), respectively, along with fetal bovine serum (Cat. NO. 10437028, GIBCO), while ensuring that the final concentration was 10%. Cells were cultured at 37 °C in a humidified incubator in a 5% CO2 atmosphere.

### 2.5. Human Papillomavirus Genotyping

Human papillomavirus genotyping of cancer tissues was carried out via the Roche Cobas 4800 HPV test. Briefly, cancer cells were stored in 800 µL Cobas PCR Cell Collection Media (Cat. No. 05619637190, Roche Molecular Systems, Inc., Branchburg, CA, USA), and Roche Cobas X 480 instruments were used to purify the DNA. Real-time PCR was performed using the Roche Cobas 4800 HPV Test on Roche Cobas Z 480 analyzers. The assay was performed and validated by the Taipei Institute of pathology, Taiwan. In addition, CTCs, isolated after negative-selection processes, were sent for HPV genotyping. All the 41 patients had p16, evidenced by immunohistochemistry staining, and had p16 CTC, evidenced by flow cytometry and genotyping.

### 2.6. Statistical Analysis

The basic characteristics of enrolled patients are demonstrated using descriptive statistics. Progression-free survival (PFS) was calculated from the date of CTC sampling, seven days before systemic chemotherapy, to cancer-specific progression or recurrence after CCRT or death from any causes. Overall survival (OS) was defined as the period from the date of CTC sampling to death from any cause. We applied chi-square and Fisher’s exact tests to determine the difference between the p16 expression status in the tissue and blood (CTCs). We also used Kaplan–Meier survival plots with the log-rank test to demonstrate the individual factors affecting survival. Patients who did not experience the event (disease progression or death) were defined to be censored in the analysis. After checking the assumptions of clinicopathological factors, we used the univariate and multivariate Cox proportional hazard regression models to identify the independent prognostic factors of PFS and OS. All potential predictor variables were analyzed in the multivariate analysis, including p16-positive and p16-negative CTC status. They are essential items in this research, although they are mutually exclusive. Statistical analysis was performed using SPSS for Windows (version 18, SPSS Inc., Chicago, IL, USA). A *p*-value of 0.05 was considered statistically significant.

## 3. Results

### 3.1. Patient Enrollment

**Figure 1:**
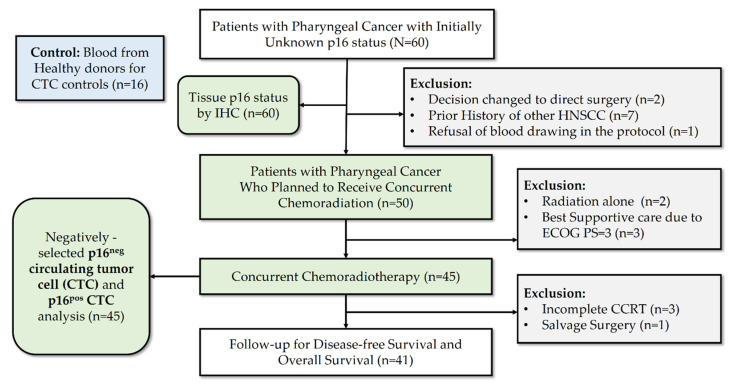
CONSORT algorithm.

A total of 76 subjects (including 16 healthy donors) were prospectively enrolled, and overall, 41 patients met all the treatment criteria and were analyzed at Chang Gung Memorial Hospital, Linkou, and at Keelung between August 2017 and August 2018. Figure 1 demonstrates the study flow and patient numbers at different stages of enrollment in this prospective study. Table 1 summarizes the characteristics of the entire population. A total of 28 (68.3%) patients had oropharyngeal cancer, while 13 (31.7%) patients had non-oropharyngeal cancer. These 13 patients were enrolled because they had initially presented with an unknown primary cancer or a huge confluent mass in the hypopharynx and oropharynx area. The median age of the cohort was 55 (37–74) years old, and 78% of the patients have relatively good performance status before the concurrent chemoradiotherapy. Totals of 19 (46.3%) and 22 (53.7%) patients tested positive and negative, respectively, for p16 expression, upon immunohistochemistry analysis. The stages of the enrolled patients were relatively advanced: 16 (39.0%) were stage IV patients. With a median follow-up time of 34.0 (3.0–44.9) months, 22 (53.7%) patients exhibited disease progression after concurrent chemoradiotherapy, and 14 (34.1%) patients died. The detection rates of p16-positive (>0 cells/mL blood) and p16-negative (≥3 cells/mL blood) CTCs were 51.2% (*n* = 21/41) and 70.7%, respectively. The cutoff values of CTCs (3 cells/mL) were the same as those used in previous studies [30,31].

### 3.2. The Identification of p16-Positive Circulating Tumor Cells in Cancer Patients

**Figure 2:**
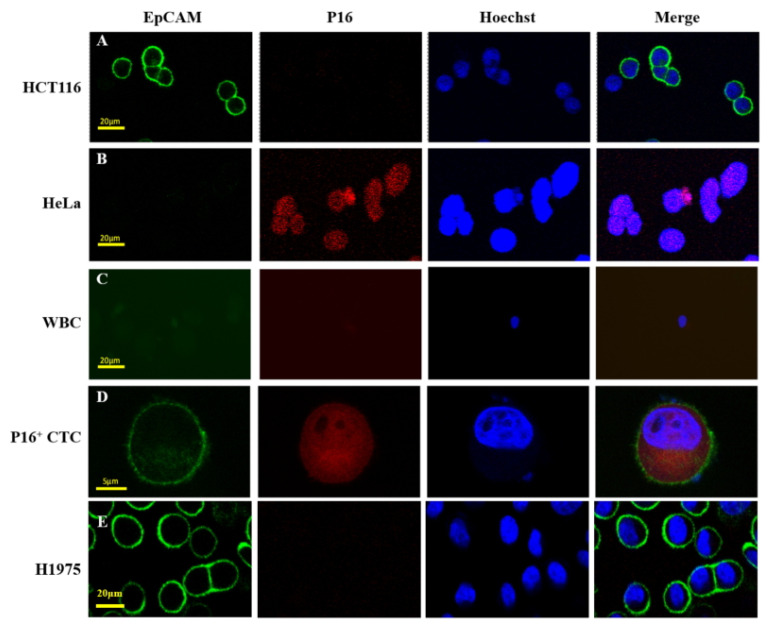
Demonstration of p16-positive circulating tumor cells identified in patients with oropharyngeal cancer. Immunofluorescence staining was used to identify cells in purified cells from blood samples. HCT116 (**A**) and HeLa (**B**) cells positively expressed EpCAM and p16, respectively. White blood cells (EpCAM^neg^/P16^neg^/Hoechst^pos^) (**C**) and p16-positive circulating tumor cells (EpCAM^pos^/P16^pos^/Hoechst^pos^) (**D**) were shown. H1975 cells (**E**) also serve as a positive control in this study. Abbreviations: CTC—circulating tumor cells.

To determine whether p16-positive CTCs could be detected in blood samples of pharyngeal cancer patients, we performed immunofluorescence staining. We used HCT116 (ATCC CCL-247)—the EpCAM^pos^p16^neg^ human colon cancer cell line—for our analysis, while HeLa (ATCC CCL-2) cells—from the EpCAM^neg^p16^pos^ human cervical cancer cell line—were used as control cells during immunofluorescence staining, as shown in Figure 2A,B. The remaining white blood cells in the CTC samples are shown in Figure 2C, while Figure 2D demonstrates the images of p16-positive CTCs, which were defined as p16^pos^- or EpCAM^pos^-nucleated cells. Otherwise, CTC without any p16 expression was be categorized as p16-negative CTCs, which have a threshold of 3 cells/mL as positive [31]. After CTC isolation, HPV genotyping was performed using commercial kits (Roche Cobas 4800 test), in accordance with the manufacturer’s instructions. Experiments involving the spiking of human blood samples with HeLa cells enabled us to identify detection limits (10 cells/2 mL human blood), as demonstrated in Supplementary Table S1.

**Figure 3:**
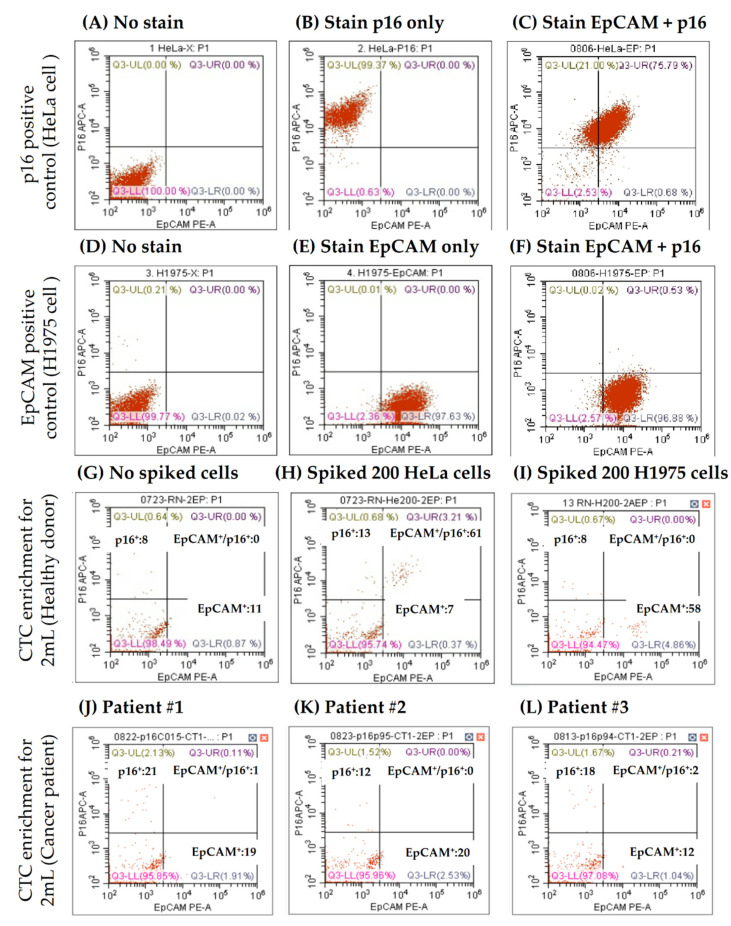
EpCAM+ and p16+ cell detection in blood samples. As standard controls, HeLa served as positive expression cells for flow cytometric analysis for p16 (**A**–**C**), and H1975 cells served as EpCAM expression (**D**–**F**). Accordingly, the protocol with controls can demonstrate p16 expression status in circulating tumor cells in one healthy individual by spiking different control cell lines (**G**–**I**) and three cancer patients (**J**–**L**).

We then applied flow cytometry-based CTC enumeration strategies and determined CTC counts after identifying the p16 expression status. In the present study, flow cytometry analysis was performed following the negative selection of CTCs, to analyze the p16-positive CTCs and p16-negative CTC in this cohort. First, HeLa (p16-positive) and H1975 (EpCAM-positive) cells were used to determine the staining conditions and set up the running template for flow cytometric analysis (Figure 3A–F). Then, tumor cell spike-in feasibility tests were carried out, as demonstrated in Figure 3G. Figure 3H,I show how p16-positive CTCs can be identified in a representative cancer patient’s blood sample. The CTC detection (≥1 cell/mL) rate was 70.7% (*n* = 29/41), and the p16 positivity rate of CTCs in the entire group was 51.2% (*n* = 21/41), irrespective of the tissue p16 status.

### 3.3. HPV Genotyping of Cancer Tissues and Circulating Tumor Cells

To illustrate the concordance of p16 expression in CTCs and tissue, we compared p16 expression levels in the tissue and blood (CTC) via IHC and flow cytometry analyses. The results showed no statistical significance (*p* = 0.155, Table 2). The results of HPV genotyping and tissue p16 expression analysis of cancer tissues were analyzed further—we found that p16 positivity on CTCs was statistically related to p16 expression or a positive HPV genotype (*p* <0.019, Table 2). However, the number of CTCs with HPV-positive genotypes was zero.

### 3.4. Effects of CTCs and p16-Positive CTCs on Survival

**Figure 4:**
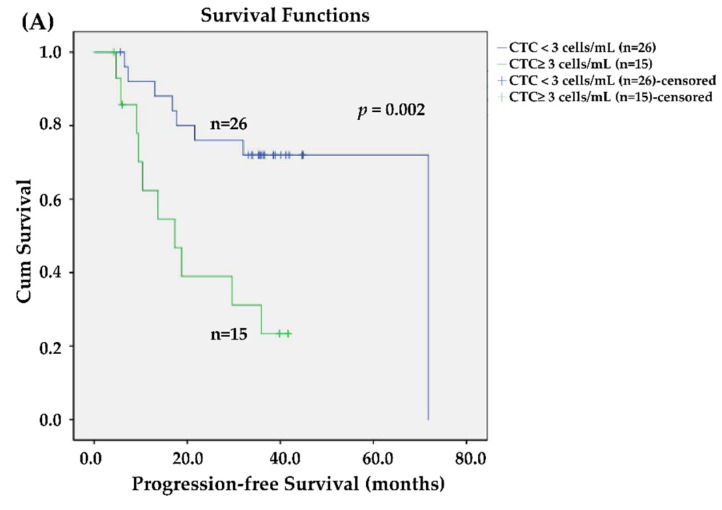

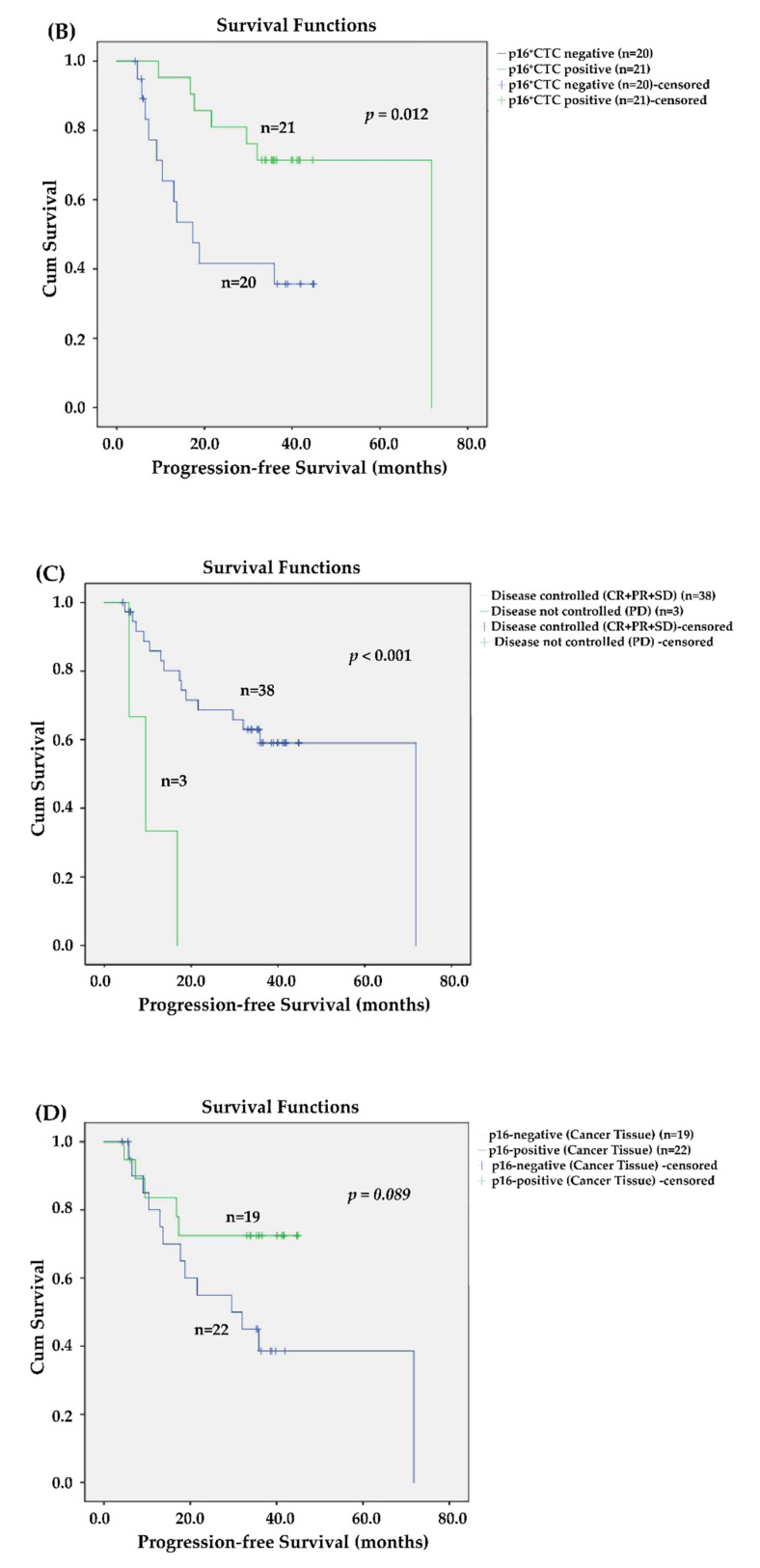

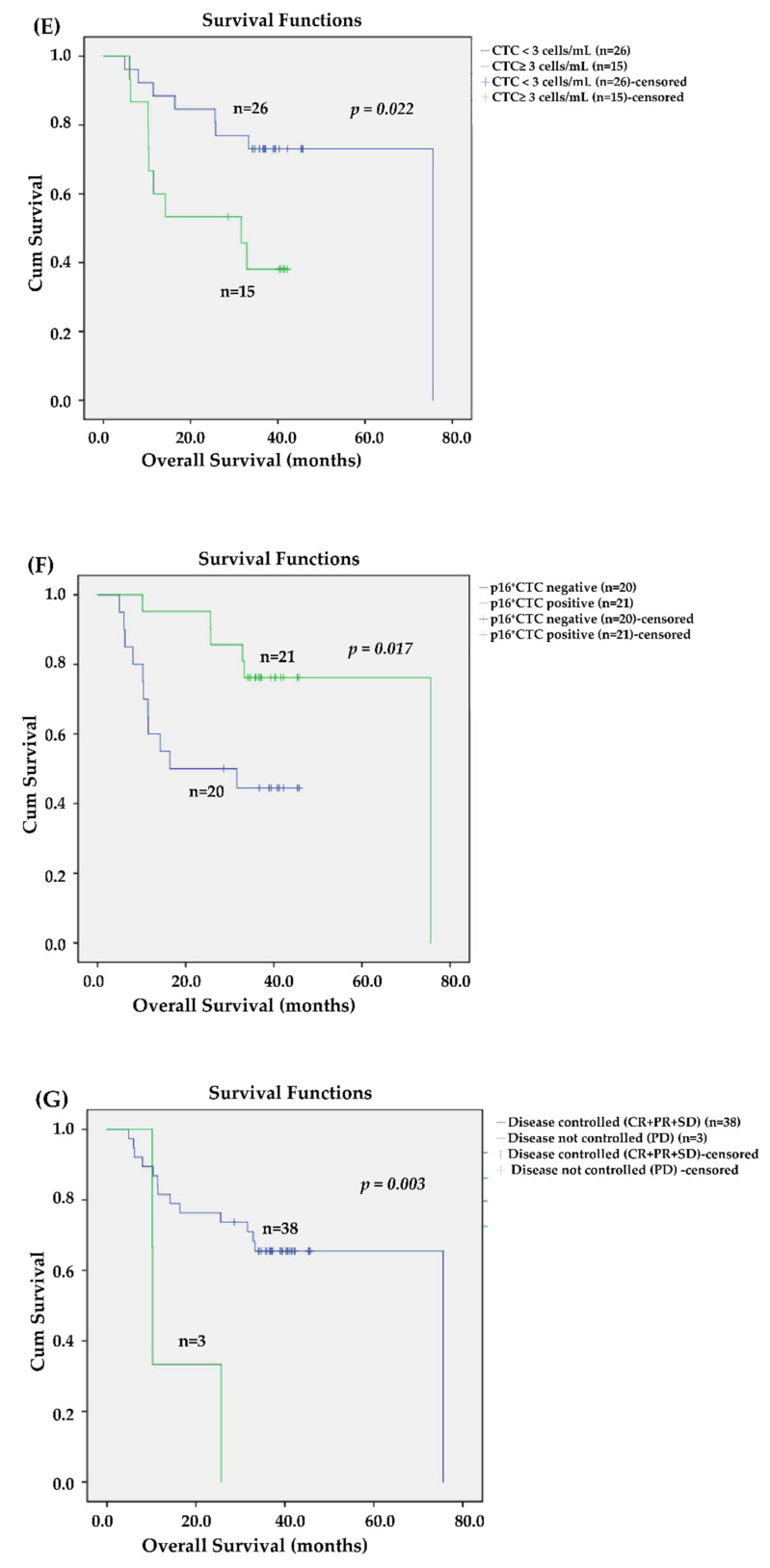

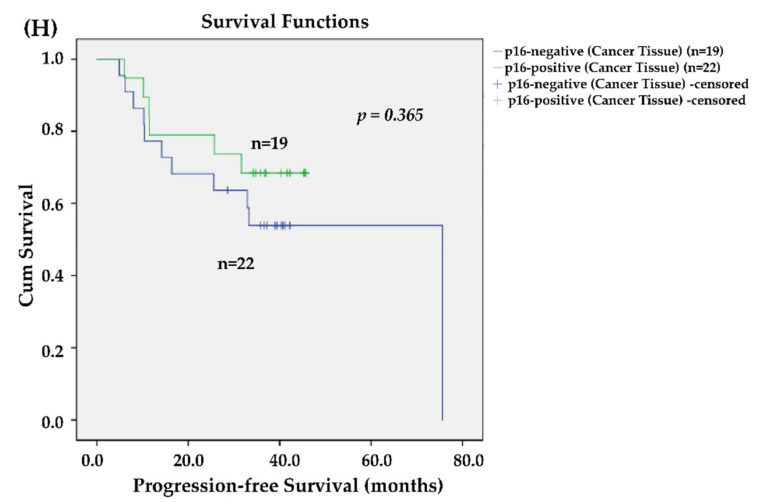
Kaplan–Meier Curves. In this cohort, patients with circulating tumor cell (CTC) numbers >=3 cells/mL had shown to negatively impact progression-free survival (PFS, *p* = 0.002) (**A**). Patients are associated with prolonged PFS, for those with p16-positive CTC (*p* = 0.012) (**B**), disease control after concurrent chemoradiotherapy was carried out (CCRT, *p* < 0.001) (**C**). However, tissue p16 expression is only marginally significant to PFS (*p* = 0.089) (**D**). For overall survival (OS), a prolonged OS is associated with patients harboring a CTC count of <3 cells/mL (*p* = 0.022) (**E**), p16 positivity of CTCs (*p* = 0.017) (**F**), and disease control after CCRT (*p* = 0.003) (**G**). Nevertheless, tissue p16 expression has no significant impact on OS in this cohort (*p* = 0.365) (**H**).

We used Kaplan–Meier survival curves to compare the factors that might influence survival in this cohort (Figure 4). Figure 4A showed that patients with CTC count ≥3 cells/mL are associated with a short PFS (*p* = 0.002). Patients with p16-positive CTCs (*p* = 0.012, Figure 4B) who exhibited disease control after concurrent chemoradiotherapy (*p* < 0.001, Figure 4C) were associated with a prolonged PFS. However, tissue p16 expression was only marginally significant for the PFS (*p* = 0.089, Figure 4D). A prolonged OS was associated with patients with CTC counts <3 cells/mL (*p* = 0.022, Figure 4E), who exhibited p16 positivity of CTCs (*p* = 0.017, Figure 4F) and disease control after CCRT (*p* = 0.003, Figure 4G). Nevertheless, tissue p16 did not affect OS in this cohort (*p* = 0.365, Figure 4H).

We analyzed all the factors in univariate and multivariate Cox regression models to identify independent prognostic factors. All factors involved in univariate analysis were analyzed during multivariate analysis using the forward LR model. Table 3 shows that the best response to CCRT, p16-negative CTC counts, and p16-positive CTCs were independent prognostic factors of disease progression, with hazard ratios (95% confidence interval) of 1.738 (95% confidence interval (CI): 1.031–2.927), 5.497 (95% CI: 1.818–16.615), and 0.176 (95% CI: 0.056–0.554), respectively. Only p16-positive CTCs (positive vs. negative) were found to be prognostic factors for cancer death, with a hazard ratio of 0.294 (95% CI: 0.102–0.852).

## 4. Discussion

This study found that p16-positive and p16-negative CTCs were uniquely correlated with survival in pharyngeal cancer patients. Our results have corroborated the results of previous studies, showing that it is feasible to detect p16-expressing CTCs [21,22]. Different methods were used to detect p16 (RT-qPCR assay [21,32,33] vs. protein expression [22]) in various studies to identify whether p16-expressing CTCs might improve risk discrimination in patients with early-stage oropharyngeal squamous cell carcinomas [21]. However, unlike the study findings by Dr. Economopoulou et al. (2019), a correlation between HPV16 E6/E7 expression in CTCs and a shorter PFS was identified in ten oropharyngeal cancer patients [21]. Hence, we hypothesized that p16-positive CTCs played a role similar to that of expressed p16 in tissues or the HPV genotyping process (Table 2 and Table 3 and Figure 4). We hypothesized that the differences might be attributable to the relatively small case number (*n* = 10) and a short period of observation in that study. Compared with other studies’ results, our study has provided relatively long-term follow-up outcomes with a median follow-up time of 34.0 months in pharyngeal cancer patients receiving CCRT. We concluded that (p16-negative) CTCs were correlated with a poor prognosis in patients with head and neck cancer: these findings were similar to those of previous studies [34,35,36,37,38,39,40].

The prognosis of patients with HPV-positive and HPV-negative head and neck cancers, especially oropharyngeal cancers, is notably different [41,42]. The p16 status of patients needs to be determined to enable clinicians to decide on the treatment plan [42,43]. Our study has provided evidence that CTC p16 positivity was independently associated with a prolonged PFS and OS. At the same time, p16-negative CTC counts were correlated with rapid disease progression after CCRT. In addition, our study has provided protocols for the identification and isolation of CTCs, and further analyzed the p16 status via a negative selection-based flow cytometric method.

We performed IHC staining and showed that the p16-positive CTC counts in flow cytometric analysis were not statistically related to the tissue p16 expression status (*p* = 0.155, Table 2) but were associated with both tissue p16 expression and HPV genotyping results (*p* = 0.019, Table 2). The main differences resulted from three cases with negative tissue IHC p16 expression but positive HPV genotyping—they were all in the p16-positive CTC group. It is well known that the discordance rate between tissue p16 expression and HPV infection by PCR could be up to 24–32% [42,44]. Some investigators have proposed that: (i) different HPV genotyping kits cannot fully identify all subtypes of HPV; (ii) the diagnostic efficacy of IHC staining p16 across countries was variable; (iii) p16 overexpression may be related to an Rb dysfunction, but Rb dysfunction may not be related to HPV infection; (iv) tumor heterogeneity or sampling bias [45,46] might cause a discrepancy between tissue p16 levels and HPV genotyping results [44]. In a meta-analysis involving 2963 patients, the IHC staining p16 expression level was more consistent with that observed during the in-situ hybridization test. It could prove to be prognostically more valuable in patients with pharyngeal cancer [45]. Our findings support that p16 expression analysis remains a cost-effective method for predicting HPV infections in daily clinical practice and makes it feasible to detect p16 expression in CTCs.

One of the most exciting findings of this study was that we found no positive HPV infection in CTC samples. We have several possible explanations for our findings. First, HPV-positive CTCs, and not p16-positive CTCs, might not intravasate into the bloodstream. Dok et al. (2017) have demonstrated that different dissemination patterns were observed in HPV-negative and HPV-positive HNSCCs because of the dual role of p16 [47]. Though p16 might impair angiogenesis, it promotes lymphatic vessel formation in patients with HPV-positive head and neck cancer [47], which might explain why patients with HPV-positive oropharyngeal cancer receive a good prognosis [48]. These findings might also explain why HPV-positive CTCs were rarely detected in the present study.

Taken together, p16-positive CTCs could provide a new risk stratification tool for diagnosis and enable us to monitor p16 expression and CTCs after curative therapy dynamically. With reference to the p16-positivity of CTCs, the de-escalation strategy in selected patients with baseline p16-positive CTCs could reduce the intensity of anticancer treatment and prevent the unnecessary physical, mental, and economic damage resulting from treatments. More importantly, a non-invasive test based on the p16 status in CTCs was able to predict the prognosis (PFS and OS) in pharyngeal cancer patients receiving CCRT. Therefore, the use of the non-invasive test could be an add-on prognostic strategy when the tissue specimen is unavailable or serial tests are required.

## 5. Limitations

The limitations of the study need to be addressed before our findings can be used in further studies or clinical practice. First, after excluding those unfit for final analysis, the sample size was relatively small (*n* = 41), which could explain why tissue p16 expression levels were not correlated to survival in the cohort. Second, the study included patients who were eventually diagnosed with hypopharyngeal cancer. This might result in some confusion during survival analysis because patients with p16-positive hypopharyngeal cancer have a different prognosis from those with p16-positive oropharyngeal cancer. The p16 expression level was found to be poorly correlated to HPV infections in patients with non-oropharyngeal cancer [12,49,50]. Although some investigators found that patients with HPV DNA and p16-positive hypopharyngeal cancer exhibited better clinical outcomes, as compared to those of patients with other types of HPV-unrelated hypopharyngeal cancers [51,52], the current consensus is that there is no clear correlation between p16 expression and survival in non-oropharyngeal cancer patients. Our findings show that, even if the data for some patients with hypopharyngeal cancer was mixed up, p16-positive CTCs and p16-negative CTCs could still play a positive role in prognosis. Third, the detection rate of HPV infection in CTCs was zero. This limitation needs to be investigated further via the analysis of HPV biology in the circulation of HNSCC patients.

## 6. Conclusions

The p16-positive and p16-negative CTCs could serve as prognostic markers for pharyngeal cancer patients receiving CCRT. A liquid biopsy might help clinicians to perform risk stratification before curative therapy and play a role in de-escalation trials in the future.

## Figures and Tables

**Table 1:** Patient characteristics (*n* = 41).

Characters	*n*	%
Age (median, range) in years	55 (37–74)	
Sex		
Female	8	19.5%
Male	33	80.5%
Tumor type		
Oropharynx	28	68.3%
Non-oropharynx ^a^	13	31.7%
ECOG PS		
0–1	32	78.0%
2	9	22.0%
Tumor stage (AJCC 8th edition) ^b^		
II	20	48.8%
III	5	12.2%
IV	16	39.0%
T classification		
T1-2	28	68.3%
T3-4	13	31.7%
Lymph node Involvement		
Negative (N0)	11	26.8%
Positive (N1-3)	30	73.2%
p16 status by IHC staining		0.0%
Negative (0–70%)	22	53.7%
Positive (>70%)	19	46.3%
CCRT completion	41	100.0%
Disease Progression after CCRT		
No	19	46.3%
Yes	22	53.7%
Cancer-related death ^c^		
No	27	65.9%
Yes	14	34.1%
Circulating tumor cells (CTCs) detection rate		
p16-positive CTCs (p16^pos^EpCAM^pos^Hoechst^pos^)	21	51.2%
p16-negative CTCs (p16^neg^EpCAM^pos^Hoechst^pos^) ^d^	20	48.8%

^a^ Patients with non-oropharyngeal cancer included 10 hypopharyngeal cancers, and 3 cancers of unknown primary site. ^b^ The staging contained p16-positive and p16-negative tumors according to AJCC 8th edition staging system. ^c^ The cancer death was updated at a median follow-up time of 34.0 (range: 3.0–44.9) months. ^d^ Circulating tumor cell counts of ≥3 cells/mL was defined positive. Abbreviations: ECOG PS—Eastern Cooperative Oncology Group performance status; AJCC—The American Joint Committee on Cancer; IHC—immunohistochemistry; CCRT—concurrent chemoradiotherapy.

**Table 2:** Comparison between tissue p16 and blood p16 expressions.

	Tissue IHC p16 Negative	Tissue IHC p16 Positive	*p*-Value	Tissue IHC p16 Negative AND HPV Genotyping Negative	Tissue IHC p16 Positive OR HPV Genotyping Positive	*p*-Value
p16^pos^ CTC Negative	13	7	0.155	13	7	0.019 *
p16^pos^ CTC Positive	9	12		6	15	

* Fisher exact test was used for the statistical significance because numbers in some cells were less than 5.

**Table 3:** Univariate and multivariate Cox regression analysis.

	Progression-Free Survival						Overall Survival					
	Univariate Analysis			Multivariate Analysis			Univariate Analysis			Multivariate Analysis *		
Factors	*p*	HR	(95% CI)	*p*	HR	(95% CI)	*p*	HR	(95% CI)	*p*	HR	(95% CI)
Age	0.135	0.957	(0.904–1.014)	0.003	0.909	(0.852–0.969)	0.338	0.973	(0.920–1.029)			
Sex (female vs. male)	0.262	2.328	(0.532–10.195)				0.371	1.967	(0.447–8.659)			
Tumor type (ORX vs. non-ORX)	0.924	0.95	(0.334–2.707)				0.415	1.524	(0.553–4.198)			
T classification (cT1-2 vs. cT3-4)	0.975	0.985	(0.374–2.592)				0.548	1.350	(0.506–3.600)			
N classification	0.421	1.214	(0.758–1.944)				0.968	0.991	(0.624–1.574)			
ECOG PS	0.171	0.377	(0.093–1.523)				0.248	0.439	(0.109–1.775)			
Best response of CCRT (Non-responders vs. responders)	0.104	1.535	(0.916–2.573)	0.038	1.738	1.031–2.927	0.114	1.499	(0.907–2.475)			
Tissue p16 IHC (Positive vs. negative)	0.099	0.415	(0.146–1.180)				0.369	0.629	(0.228–1.731)			
p16^neg^ CTC (Positive vs. negative)	0.005	4.029	(1.522–10.668)	0.003	5.497	1.818–16.615	0.029	3.037	(1.123–8.213)			
p16^pos^ CTC (Positive vs. negative)	0.018	0.300	(0.110–0.816)	0.003	0.176	0.056–0.554	0.024	0.294	(0.102–0.852)	0.024	0.294	(0.102–0.852)

* All factors in the univariate analysis were examined in the multivariate model. Abbreviations: CTC—circulating tumor cells; AJCC—The American Joint Cancer Committee; ECOG PS—Eastern Cooperative Oncology Group performance status; CCRT—concurrent chemoradiotherapy; PD—progressive disease; SD—stable disease; PR—partial response; CR—complete remission; IHC—immunohistochemistry; HR—hazard ratio; CI—confidence interval.

## Data Availability

All the data are available on demand.

## Supplementary Materials

The following are available online at https://www.mdpi.com/article/10.3390/jpm11111156/s1, Table S1: the limits of human papillomavirus genotyping detection on blood samples.

## Abbreviations

**Full name**	**Abbreviations**
head and neck squamous cell carcinomas	HNSCC
human papillomavirus	HPV
circulating tumor cells	CTCs
confidence interval	CI
concurrent chemoradiotherapy	CCRT
American Joint Committee on Cancer	AJCC
response evaluation criteria in solid tumors	RECIST
complete remission	CR
partial response	PR
Stable disease	SD
progressive disease	PD
Immunohistochemistry staining	IHC
Progression-free survival	PFS
Overall survival	OS
Eastern Cooperative Oncology Group performance status	ECOG PS
