# Supplementary material for: Circulating p16-Positive and p16-Negative Tumor Cells Serve as Independent Prognostic Indicators of Survival in Patients with Head and Neck Squamous Cell Carcinomas
Source: J Pers Med. 2021 Nov 7;11(11):1156. doi: 10.3390/jpm11111156 (PMC8624430; doi:10.3390/jpm11111156)
Supplement: Supplementary file 1 [file jpm-11-01156-s001.zip › jpm-1427888-supplementary.pdf]

Supplementary Data

Supplementary Table S1. The limits of human papillomavirus genotyping detection on blood samples

Sample types	Test groups	Repeats of HPV genotyping tests
**Cell mixture (HeLa cells + WBC)**	10^3 HeLa	+/+/+ (n=3)
	10^4 WBC	-/-/- (n=3)
	10^3 HeLa+ 10^4 WBC	+/+/+ (n=3)
	10^2 HeLa+ 10^4 WBC	+/+/+ (n=3)
	10 HeLa+ 10^4 WBC	+/+/+ (n=3)
**Spike test (HeLa cells in 2ml human blood from healthy donors)**	10^3 HeLa	+/+/+/+ (n=4)
	10^2 HeLa	+/+/+/+ (n=4)
	20 HeLa	+/+/+/+ (n=4)
	10 HeLa	+/+/+/+ (n=4)
	5 HeLa	-/-/-/+ (n=4)
	0 HeLa	-/-/-/- (n=4)

Abbreviations: HPV, human papillomavirus; +, indicates one positive result of HPV detection test and +/+/+ means 3 repeated experiments showed all positive findings of HPV infection.
